# Design and Synthesis of a Chitodisaccharide-Based Affinity Resin for Chitosanases Purification

**DOI:** 10.3390/md17010068

**Published:** 2019-01-21

**Authors:** Shangyong Li, Linna Wang, Xuehong Chen, Mi Sun, Yantao Han

**Affiliations:** 1 Department of Pharmacology, College of basic Medicine, Qingdao University, Qingdao 266071, China; chen-xuehong@163.com; 2 Key Laboratory of Sustainable Development of Marine Fisheries, Ministry of Agriculture, Yellow Sea Fisheries Research Institute, Chinese Academy of Fishery Sciences, Qingdao 266071, China; wlnwfllsy@163.com (L.W.); sunmi0532@yahoo.com (M.S.)

**Keywords:** chitosanases, adsorption analysis, affinity purification

## Abstract

Chitooligosaccharides (CHOS) have gained increasing attention because of their important biological activities. Enhancing the efficiency of CHOS production essentially requires screening of novel chitosanase with unique characteristics. Therefore, a rapid and efficient one-step affinity purification procedure plays important roles in screening native chitosanases. In this study, we report the design and synthesis of affinity resin for efficient purification of native chitosanases without any tags, using chitodisaccharides (CHDS) as an affinity ligand, to couple with Sepharose 6B via a spacer, cyanuric chloride. Based on the CHDS-modified affinity resin, a one-step affinity purification method was developed and optimized, and then applied to purify three typical glycoside hydrolase (GH) families: 46, 75, and 80 chitosanase. The three purified chitosanases were homogeneous with purities of greater than 95% and bioactivity recovery of more than 40%. Moreover, we also developed a rapid and efficient affinity purification procedure, in which tag-free chitosanase could be directly purified from supernatant of bacterial culture. The purified chitosanases samples using such a procedure had apparent homogeneity, with more than 90% purity and 10–50% yield. The novel purification methods established in this work can be applied to purify native chitosanases in various scales, such as laboratory and industrial scales.

## 1. Introduction

Chitosan, a natural cationic polysaccharide, has key roles in many biological processes, such as artificial skin, absorbable surgical sutures, and wound healing accelerators [1]. Chitosanase (EC 3.2.1.132) catalyzes the hydrolysis of β-1,4-linked glycosidic bond in the chitosan chain, releasing chitooligosaccharides (CHOS) as products [2]. In recent years, CHOS have increasingly gained more attention because of their important biological activities, such as anti-tumor [3,4], immuno-enhancing [5], anti-fungi [6], anti-bacterial [7], and anti-diabetic effect [8]. These activities are dependent on chemical structures and molecular sizes of the oligosaccharides [9].

It has been identified that chitosanases can be isolated from various organisms, including fungi, plants, and bacteria [10,11]. Based on the classification in Carbohydrate-Active enZYmes (CAZy) databases [12] (http://www.cazy.org), chitosanases are included into seven different glycoside hydrolase (GH) families: 3, 5, 7, 8, 46, 75, and 80. Currently, most known chitosanases belong to GH family 46, which comprise only chitosanases. Thus far, several chitosanases from different organisms have been identified [13,14,15,16]. According to the sequence alignments, chitosanases are classified into seven GH families: 3, 5, 7, 8, 46, 75, and 80 [11]. Among which, chitosanases, which belong to GH family 46, have been fully characterized, and several crystal structures of chitosanases have been determined (PDB codes: 1CHK, 1QGI, and 2D05). The catalytic mechanism of chitosanases from GH family 46 has been elucidated; it was identified to follow an “inverting” catalytic mechanism [12,13,14]. The 3-D crystal structure of chitosanase-substrate complex (CsnOU01) shows that the −2, −1, and +1 subsites of chitosanase from GH family 46 play a predominant role for the formation of hydrogen bond intermediate during substrate binding and catalysis [12,13]. Because chitosanases with special characteristics have potential applications in industry and biotechnology, a rapid and efficient purification method that can be used to purify chitosanases, from which their biochemical characteristics are determined, from different microorganisms is essential. According to the literature, the purification protocols of native chitosanases generally involve ultrafiltration, ammonium sulfate precipitation, salting out, hydrophobic interaction chromatography, ion-exchange chromatography, or gel filtration chromatography [13,14,15,16]. These traditional methods not only require a large number of steps, but are also time-consuming and difficult to scale up. Biomimetic affinity chromatography is potentially the most selective method in protein purification [17,18]. This technique requires lower number of steps, while resulting in higher yields, so can be considered economical. Therefore, it can be beneficial to develop a rapid and efficient one-step affinity purification protocol for chitosanase.

Affinity purification of enzymes often design affinity ligands that function as substrate analogues or specific inhibitors [16,18,19]. In this study, CHOS-based resin was synthesized by coupling chitosan-disaccharide (CHDS) to epoxy-activated Sepharose 6B using cyanuric chloride as a spacer. The resin was then used in the development of one-step affinity purification of chitosanases, in which three typical enzymes, GH families 46, 75, and 80, were purified. The developed purification protocol was highly efficient and resulted in high purity enzymes (more than 95% purity). The method was also applied to directly purify chitosanase from bacterial culture medium.

## 2. Results and Discussion

### 2.1. Design and Synthesis of CHOS-based Affinity Resin

**Figure 1:**
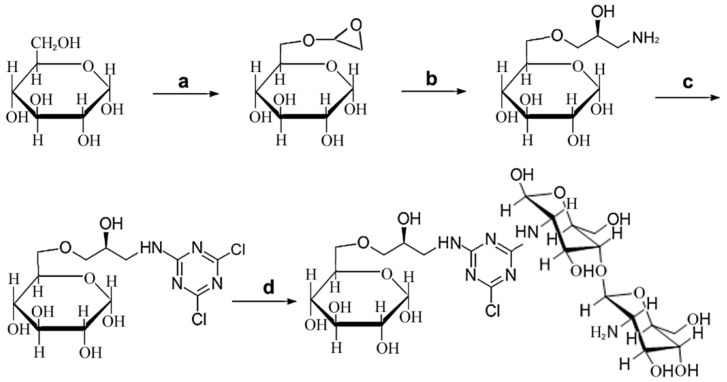
Synthesis protocol and scheme of the CHDS ligand coupled with active Sepharose 6B via cyanuric chloride spacer. Reagents and conditions: (**a**) epichlorohydrin, DMSO, NaOH aqueous solution, 2.5 h; (**b**) 35% saturated ammonia, overnight; (**c**) cyanuric chloride, 50% acetone, pH 7–8; (**d**), CHDS, sodium carbonate, 24 h.

**Figure 2:**
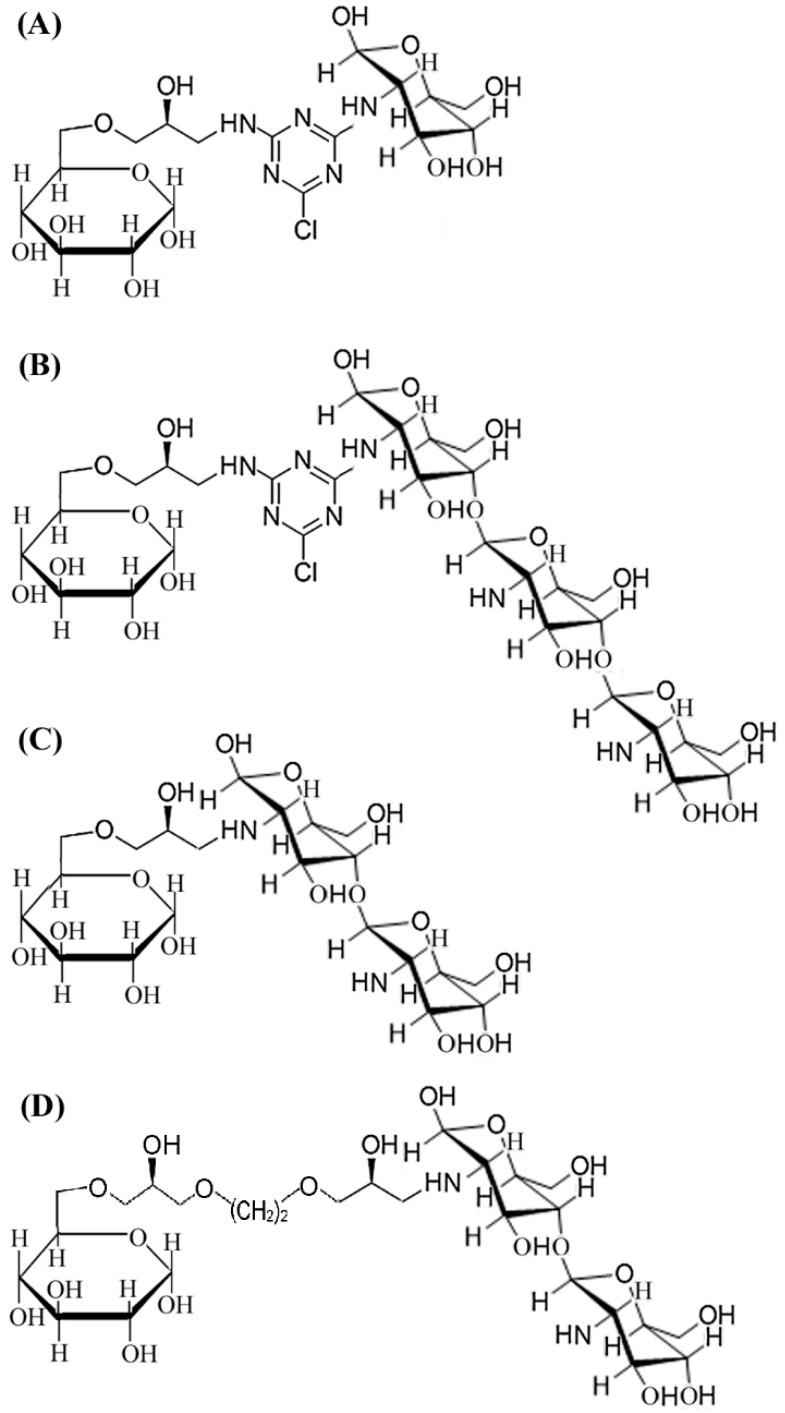
Schemes of four Sepharose 6B resins with different ligand and spacer. (**A**) Glucosamine ligand via cyanuric chloride spacer. (**B**) CHTS ligand via cyanuric chloride spacer. (**C**) CHDS ligand via 5-atom spacer arm. (**D**) CHDS ligand via 10-atom spacer arm.

Reversible inhibitor or substrate analogue are commonly used for the design of biomimetic affinity ligands of enzymes, due to the mild and efficient affinity values [17]. Chitooligosaccharides (CHOS) are natural cationic saccharides, while the catalytic domain of chitosanases is rich in acidic amino acids [12,13]; the acid-base interactions between the two molecules can provide affinity force during affinity purification. Immobilization of a ligand onto epoxy-activated resin can be achieved via nucleophilic groups (often is primary amine) presented in the ligand [14,19,20,21,22]. Because CHOS contains an amine group at the C-2 position of the sugar ring, we thus focused our efforts on the design of CHOS-based affinity resin for purification of chitosanase. The natural properties of affinity resins—in other words the selection of affinity ligands and spacers—have an important impact on the results of biomimetic affinity purification [19]. As reported previously, the final degradation products of the endo-type chitosanase are CHDS and chitotrisaccharide (CHTS), whereas that of the exo-type chitosanase is glucosamine [11]. To obtain affinity resin with optimal ligands, two types of CHOS-based ligands, CHDS-based and CHTS-based, were compared, and glucosamine was also chosen as a contrasted affinity ligand. The scheme for the synthesis protocol of CHDS-based Sepharose 6B is shown in Figure 1. The CHTS-based resin and glucosamine-based resin were synthesized from CHTS or glucosamine at the same concentration as that of CHDS (Figure 2A,B). The ninhydrin test was applied to examine the density of the free amino group (Table 1), and the linkage of cyanuric chloride to the amino groups. Purple color indicated the presence of free amino groups, and color disappearance indicated that cyanuric chloride had been linked to the amino groups. Through the change of purple color, almost all of the free amino groups linked to the cyanuric chloride. A ninhydrin test was also used to determine the coupling efficiencies and yields for CHDS, which showed an extra free amino group. About 16.8 μmol/ml free amino group was determined by ninhydrin test. The yields of the final affinity product were about 80.3%.

**Figure 3:**
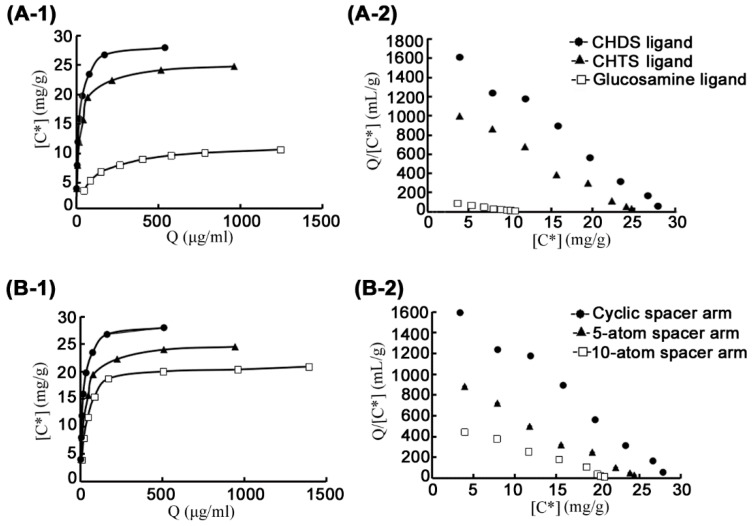
Adsorption analyses of GH family 46 enzyme CsnOU01. (**A**) Adsorption analysis of affinity resins with different ligands via cyanuric chloride as a spacer arm. (**B**) Adsorption analysis of affinity resins with CHDS as affinity ligand via different spacer arms. (**1**) Equilibrium adsorption of enzyme and affinity resin. (**2**) Plot describing the equilibrium of the absorption on the resin and the enzyme concentration in the liquid phase.

In this study, the typical GH family 46 chitosanase, CsnOU01, was chosen as the target protein in the determination of equilibrium adsorption of different affinity resins (Figure 3). The densities of free amino groups were determined by the ninhydrin test before the addition of the affinity ligand, giving equal ligand densities (Table 1). To find the optimal affinity ligand, control resins were synthesized from CHTS or glucosamine according to the method described above (Figure 2A,B). Equilibrium adsorption studies were performed to characterize the affinity value of CsnOU01 and these three affinity media (Figure 3A). The adsorption constant for CHDS-based resin was 16.4 μg/mL, which was notably lower than that for CHTS-based resin (20.7 μg/mL) and glucosamine-based resin (88.5 μg/mL). Additionally, the theoretical maximum absorption (*Q*_max_) for the CHDS-based resin was significantly higher than that for other two types of resins (Table 1), indicating that the affinity of CHDS-based resin is high. Therefore, CHDS was chosen as the affinity ligand for further design and synthesis of affinity resins.

To find the optimal spacer arm length, cyclic arm (cyanuric chloride) and linear arms (5-atom length and 10-atom length) were compared. Cyanuric chloride (2,4,6-trichloro-1,3,5-triazine) is a compound containing *s*-triazine (C_3_N_3_) ring, which can exert higher strength for ligand stabilization; it is widely used in the synthesis of affinity resin [23]. Figure 2C,D showed the corresponding scheme for the synthesis of resins, with spacers of 5-atom and 10-atom lengths, are shown in Figure 2C,D. According to the adsorption analysis (Figure 3B), CHDS ligand with cyclic spacer arm exhibited the highest desorption value (*K*_d_, 16.4 μg/mL; *Q*_max_, 30.9 mg/g), with an epoxy content (20.9 µmol/mL) lower than the content of 5-atom linear spacer and 10-atom linear spacer (Table 1). Therefore, cyanuric chloride was chosen as the optimal spacer arm. These observations indicate that in addition to CHDS ligand, the cyanuric chloride spacer arm is also important for the binding to chitosanase. Thus, in the larger scale of resin synthesis and chitosanases purification, CHDS was used as a ligand to couple with Sepharose 6B affinity resin through a spacer arm cyanuric chloride.

### 2.2. Affinity Purification of Chitosanases from Different GH Families

Three chitosanases (CsnOU01, Csn, and ChoA) from GH families 46, 75, and 80, respectively, were expressed in *E. coli* BL21(DE3) through pET-22b(+) system, with or without 6×His tag. After centrifugation at 12,000 rpm for 10 min, the supernatant containing strains without 6×His tag was loaded onto a 10 mL pre-equilibrated column and then washed with washing buffer (0.1 M Tris-HCl buffer, pH 8.0) until the eluate exhibited no detectable absorbance at 280 nm. Thereafter, the enzymes were purified by the established one-step purification using CHDS-based Sepharose 6B resin.

We tested different loading and elution conditions to optimize the yield of chitosanases (Supplementary Table S1). Chitosanases are stable at a pH range of 4.0–8.0, and *Q*_max_ values are usually determined at pH 8.0 with Tris-HCl buffer; therefore, 0.1 M Tris-HCl, pH 8.0 was used as the loading buffer. In previous elution process, non-target proteins were depleted by 0.1 M Tris-HCl, pH 8.0 containing 100 mM NaCl, in which the target protein was not eluted. In optimization of elution pH, elution buffers containing acetic acid buffers with different pH, ranging from 4.0–6.0, were used in the elution of chitosanases, and the results were compared (low pH buffers are known to favor the disruption of H-bond between chitosanases and affinity-based resin, especially for the substrate analogue-based resin). The highest protein yield was obtained at pH 5.4; 0.1 M acetic acid buffer, pH 5.4 was thus chosen as the elution buffer. In addition, the elution buffer was supplemented with 0.8 M NaCl to further deteriorate the affinity between the enzyme and the resin.

**Figure 4:**
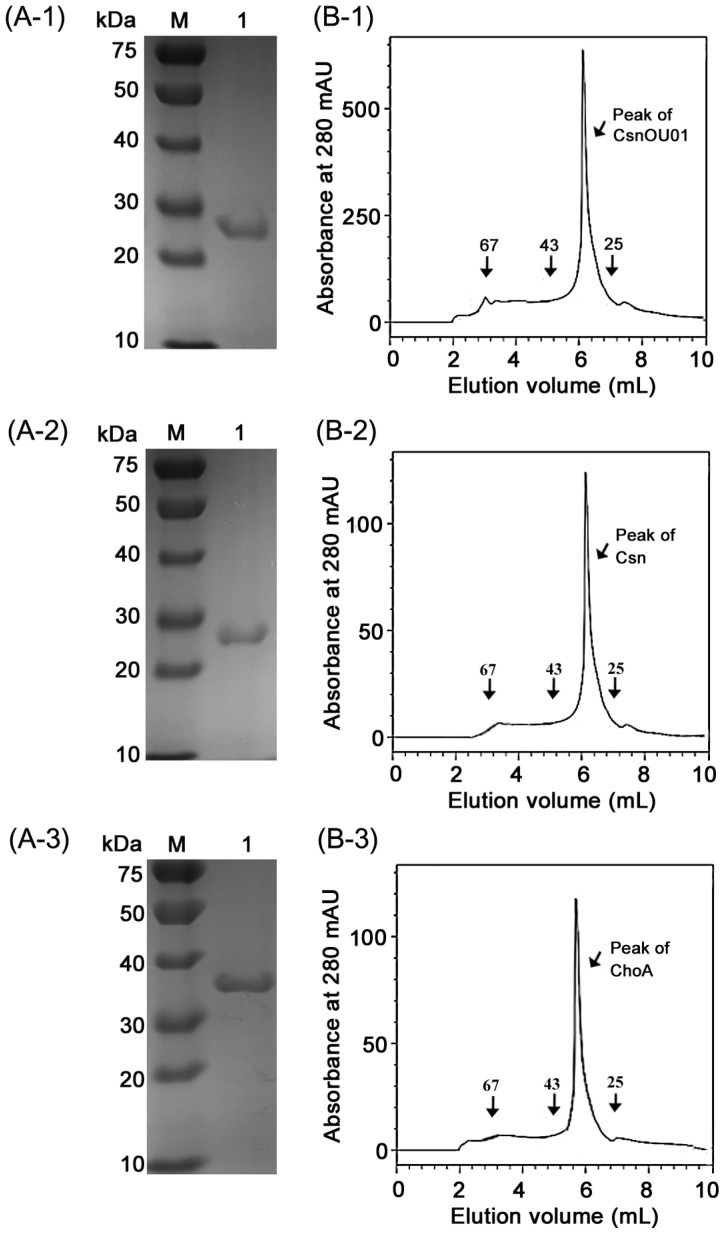
Purity analysis of purified chitosanases. (**A**) SDS-PAGE analysis of purified enzymes. *Lane M*, molecular mass standard protein marker. (**A-1**) the purified CsnOU01. (**A-2**) the purified Csn. (**A-3**) the purified ChoA. (**B**) HPLC analysis of the purified CsnOU01 (**1**), Csn (**2**), and ChoA (**3**) on a TSK 3000 SW column.

The established one-step purification method took a total time of as low as 10 min at the flow rate of 3 mL/min. With this simple and efficient affinity chromatography, CsnOU01 was purified with purities of ~98 and 98.7% according to SDS-PAGE (Figure 4(A-1)) and HPLC analysis (Figure 4(B-1)), respectively. The purification yielded CsnOU01 of 5.4 folds with the specific activity of 356.8 U/mg, and the molecular mass of the purified CsnOU01 was determined to be ~28 kDa, which was in good agreement with the theoretical molecular mass [24,25]. The bioactivity yield as a result of this affinity purification method was about 64.1% (Table 2). To determine whether or not the synthesized affinity resin has affinity for chitosanases from other GH families, Csn from GH family 75 [26] and ChoA from GH family 80 [13] were purified using the synthesized resin. As shown in Table 2, CHDS-based resin could efficiently purify the two chitosanases, with bioactivity recoveries of 45.2% and 40.8% for Csn and ChoA, respectively. The analysis by SDS-PAGE (Figure 4(A-2,A-3)) and HPLC using size-exclusion chromatography (Figure 4(B-2,B-3)) showed that both enzymes had purities of more than 95%.

The traditional purification protocol towards CsnOU01, Csn, and ChoA was developed and shown in Supplementary Table S2. Here, we compared CHDS-based affinity protocol with the traditional methods, reporting in Table 2 all the different purification steps, activity yields, and specific activities of pure enzyme. The traditional protocol with multiple steps is expensive and leads to low recoveries. The specific activity of enzymes purified by the CHDS-based protocol and the traditional purification protocol is similar. However, the purity of CHDS-based affinity purification is higher than the traditional methods.

As a contrast, Ni-NTA Sepharose 6B resin was also used to purify the three recombinant chitosanases containing 6×His-tag, by immobilized metal affinity chromatography (IMAC). Even if the IMAC protocol led to an activity recovery higher than the CHDS-based affinity protocol, the specific activities are lower (Table 2). Because of all the obvious advantages of the CHDS-based affinity protocol, including one-step chromatography, no use of toxic imidazole, higher purity, and shorter times, this approach has the potential to be used for industrial applications of high purity chitosanase.

### 2.3. Direct Purification of Chitosanases from Bacterial Culture Medium

**Figure 5:**
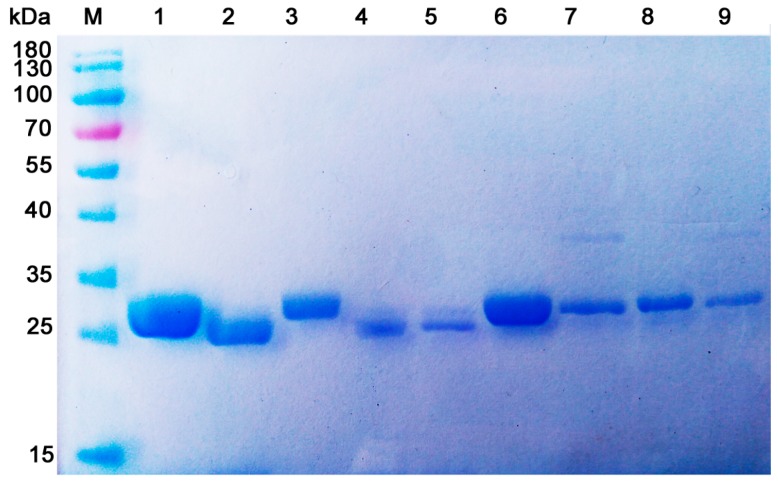
SDS-PAGE analysis of purified chitosanases direct from bacterial supernatant.

In order to establish a rapid purification protocol for native chitosanase, nine bacterial strains with high chitosanase activity (more than 50 U/mL) were chosen as target strains in the affinity purification of chitosanase using the established CHDS-based materials. After bacterial culture was centrifuged at 10,000× *g* for 15 min, the supernatant was loaded onto 10 mL pre-equilibrated column and washed with washing buffer (0.1 M Tris-HCl buffer with 100 mM NaCl, pH 8.0) until the eluate showed no detectable absorbance at 280 nm. After that, the target protein was eluted with elution buffer (0.1 M acetic acid buffer, pH 5.6, 0.8 M NaCl). As shown in Figure 5, CHDS-based resin was able to purify chitosanases (with certain homogeneity) from culture medium.

The activity recovery and purity of the purified enzymes were shown in Table 3. The purified chitosanase samples using such procedure had apparent homogeneity with more than 90% purity and 10–50% yield. Different strains showed different activity recoveries. This result may be caused by multi-factors. In this study, our affinity purification condition was only used for rapidly screening chitosanases. The optimal purification protocol toward the special enzyme needs further optimization. After characterizing these purified enzymes, the chitosanase from *Serratia* sp. QD07 showed high thermo-tolerant property and suitability for industrial usage (data not shown). The characterization data of this thermo-tolerant enzyme will be reported in the next paper.

Chitosanases that have special characteristics can potentially be applied in biotechnology industry and other fields. As has been described in the literature, the purification of native chitosanases (without His-tag) usually involves ultrafiltration, ammonium acetate precipitation, salting out, ion-exchange chromatography, and hydrophobic interaction chromatography [13,14,15,16]. These conventional methods usually involve a large number of steps, which are time-consuming and difficult to scale up. As far as we know, biomimetic affinity chromatography specially designed for native chitosanase has not been established. In this study, we developed the rapid and efficient affinity purification procedure, in which native chitosanase could be directly purified from supernatant of bacterial culture. The novel purification methods established in this work can be applied to screen and purify chitosanases, both in laboratory and industrial scales.

## 3. Materials and Methods

### 3.1. Materials

Chitosan, with degree of deacetylation (DDA) ≥ 95%, was purchased from Aladdin, China. To obtain chitodisaccharide (CHDS) and chitotrisaccharide (CHTS), chitosan (0.1%) was degraded by GH family 46 chitosanase CsnOU01 at a final enzyme concentration at 20 mg/mL for 6 h. CHDS and CHTS were purified from the degradation products of chitosan using a Biogel P-2 column (GE Healthcare, Madison, WI, USA). Briefly, 100 mg degradation product of CHDS and CHTS was loaded into the Biogel P-2 column (1.6 × 130 mm), using 0.2 M ammonium acid carbonate as mobile phase. The flow rate was 1 mL/min. Then, the effluent was collected every 1 min and the sugar content was determined by phenol sulfate method. Finally, oligosaccharides were collected and identified by TLC. Sigma-Aldrich (St. Louis, MO, USA) provided cyanuric chloride (2,4,6-trichloro-1,3,5-triazine) and glucosamine. Beijing Weishibohui Chromatography Technology Co., China, provided activated Sepharose 6B with two different spacer arm lengths (5-atom and 10-atom). Sinopharm Chemical Reagent (Shanghai, China) provided other analytical grade reagents.

### 3.2. Synthesis of Affinity Resins

CHDS-based affinity resins were synthesized according to our previous published method [20,21,27]. The synthesis scheme is shown in Figure 1. Originally, activated amino-sepharose resins were formed by modifying Sepharose 6B (100 g) using epichlorohydrin (Figure 1a). Briefly, Sepharose 6B (100 g) was first thoroughly washed with distilled water at a 10:1 ratio. After being drained and aired, Sepharose 6B was suspended in 50 mL activating solution (0.8 M NaOH aqueous solution, containing 25% DMSO and 10 mL epichlorohydrin) for 2 h at 40 °C. To form aminated Sepharose 6B, activated Sepharose 6B was suspended in 350 mL of distilled water and 35% saturated ammonia was added (150 mL) for mixing. The mixture was incubated for 6 h at 30 °C on a rotary shaker (Figure 1b). After that, cyanuric chloride (2,4,6-trichloro-1,3,5-triazine) was linked as a scaffold for the amino groups. The mixture was shaken in ice-salt bath, then 8 g of cyanuric chloride (44 mmol from Sigma-Aldrich; St. Louis, MO, USA), dissolved in 350 mL acetone, was slowly added (Figure 1c). About 100 mL NaOH aqueous solution (1 M) was slowly added to maintain the neutral pH. To clear away free cyanuric chloride, 50% (*v/v*) acetone was utilized to wash the resins. The ninhydrin test was applied to examine the density of free amino groups and the linkage of cyanuric chloride to the amino groups, according to the previously described procedure [18,20,21]. Briefly, a small aliquot of gel was smeared on filter paper, sprayed with ninhydrin solution (0.2%, *w/v*, in acetone), and heated briefly with a hair dryer. Purple color indicated the presence of free amino groups and color disappearance indicated that cyanuric chloride had been linked to the amino groups. Subsequently, dichlorotriazinylated Sepharose 6B resins were added with two-fold molar excess of CHDS dissolved in 2 M sodium carbonate and stirred for 24 h at room temperature (Figure 1d). The coupling efficiencies and yields for the CHDS ligands were also determined by ninhydrin test. Control resins were synthesized from chitotrisaccharide (CHTS) or glucosamine, according to the method described above (Figure 2A,B). Control resins with 5-atom or 10-atom spacer arms were also synthesized from CHDS-modified Sepharose 6B resins, according to the previously published method [21]. The schemes are shown in Figure 2C,D.

### 3.3. Expression and Purification of Three Typical Chitosanase

Currently, GH families 46, 75, and 80 comprise only chitosanases in the CAZy database [10,11,28]. Genes encoding three typical chitosanases, including CsnOU01 from GH family 46 (Genbank number ABM91442), Csn from GH family 75 (Genbank number AFG33049), and ChoA from GH family 80 (Genbank number BAA32084) were cloned into the pET22b (containing 6×His tag) vector and expressed in *Escherichia coli* BL21(DE3). The genes were optimized for *E. coli* and synthesized by BGI (Qingdao, China). The DNA fragment was digested to introduce *Nco* I and *Xho* I sites, then ligated into the *Nco* I and *Xho* I sites of plasmid pET22b. The recombinant plasmid was transferred into *E. coli* BL21 (DE3). Cells were cultured in LB medium containing 30 μg/mL ampicillin at 37 °C, until the OD600 reached 0.6. Afterwards, the expression of the target gene was induced by 0.1 mM isopropyl-β-thiogalactoside (IPTG) at 20 °C and 100 rpm for 18 h. These chitosanases with 6 his-tags were purified using a Ni-NTA Sepharose 6B column (GE Healthcare, Madison, WI, USA) at an AKTA avant 150 platform. After centrifugation for 10 min at 12,000 rpm, the supernatant was loaded into 10 mL equilibrated affinity column and washed with washing buffer (0.1 M Tris–HCl buffer, pH 7.6) until the elute exhibited no detectable absorbance at 280 nm. Then, the elution buffer 1 (0.02 M Tris-HCl, pH 7.6, with 10 mM imidazole) was used to deplete the impure protein. The target protein was eluted by elution buffer 2 (0.02 M Tris-HCl, pH 7.6, with 150 mM imidazole). The flow rate of the mobile phase was 3.0 mL/min. The concentrations of each elution peak were assayed by the Bradford method, using BSA as a standard. Chitosanses without 6 his-tag were purified by the traditional method and CHDS-based method, as shown in Section 3.5. Molecular weight and purity of the enzymes were confirmed by SDS-PAGE or HPLC with size-exclusion chromatography.

### 3.4. Calculation of Desorption Constant of Chitosanase

The characterization of the interactions between chitosanases and affinity resins was carried out using equilibrium adsorption study. Scatchard analysis model was used for analysis of the desorption constant (*K*_d_) and the theoretical maximum adsorption capacity (*Q*_max_) of different affinity resins [22,29]. Various concentrations of enzymes (10 mL, 0.1–0.9 mg/mL in 20 mM Tris-HCl buffer, pH 8.0) were combined with 5 g of each type of resin to reach the adsorption equilibrium in a shaken condition. Mixed culture was subsequently centrifuged at 1500× *g* for 5 min at 4 °C. Afterward, the residual activity of chitosanase and protein concentration in the supernatants was measured and analyzed according to the following Equation:

(1) $$Q=\frac{Q_{\max}\left[C\;*\right]\;}{K_{d}+\left[C\;*\right]\;}$$

where *Q* represents the amount of chitosanase adsorbed to the affinity resin (mg/g wet resin), *Q*_max_ represents the theoretical maximum absorption of chitosanase to the affinity resin (mg/g wet resin), [C*] represents the protein concentration of chitosanase in the mixed solution (mg/mL), and *K*_d_ represents the desorption constant. Scatchard plot represents one of the linearized forms of Equation (1). Equation (1) could be transformed into the following Equation (2):

(2) $$\frac{Q}{\left[C\;*\right]\;}=\frac{Q}{K_{d}}+\frac{Q_{\max}}{K_{d}}$$

According to the Scatchard model, a plot of *Q*/[C*] against *Q* should yield a straight line. The batch adsorption of CsnOU01 towards the affinity medium showed that the respective correlation coefficient R^2^ ranged from 0.921 to 0.991. These results indicate that the data fit well with the model.

### 3.5. Affinity Purification of Chitosanases Using CHDS-Based Resin

The synthesized CHDS-based affinity resins were pre-equilibrated with sample loading buffer (0.1 M Tris-HCl buffer, pH 8.0). Before sample was loaded, the supernatant of expression strains were centrifugated at 10,000× *g* for 10 min to remove the impurity. Approximately 100 mL samples were loaded onto 10 mL column with synthesized resins. Next, the column was washed with 30 mL washing buffer (0.1 M Tris-HCl buffer with 100 mM NaCl, pH 8.0). Elution buffers (0.1 M acetic acid buffer, pH 5.6, 0.8 M NaCl) were used for eluting the target proteins. The flow rate was 5.0 mL/min. As a control, the traditional purification protocols of three different chitosanses were also developed. The traditional purification protocol of CsnOU01 (GH46) was composed of five steps, including ultrafiltration with a Millipore Amicon^®^ Ultra-10 kDa in 15 mL filter, 60% saturation ammonium sulfate precipitation in an ice-bath and still stirring for more than 2 h, desalting with a 5 mL desalting column (GE Healthcare, Madison, WI, USA), DEAE anion-exchange chromatography, and superdex 75 gel-filtration chromatography. The traditional purification protocol of Csn (GH75) was composed of three steps, including 60% saturation ammonium sulfate precipitation in an ice-bath and still stirring for more than 2 h, desalting with a 5 mL desalting column (GE Healthcare, Madison, WI, USA), and DEAE anion-exchange chromatography. The traditional purification protocol of ChoA (GH80) was composed of six steps, including 40% saturation ammonium sulfate precipitation in an ice-bath and still stirring for more than 2 h, phenyl hydrophobic chromatography (GE Healthcare, Madison, WI, USA), desalting with a 5 mL desalting column, DEAE anion-exchange chromatography, Superdex 75 gel-filtration chromatography, and Superdex 200 gel-filtration chromatography. Protein concentrations of elution peaks were determined by Bradford assay. The purified enzymes were analyzed by 10% SDS-PAGE and HPLC with size-exclusion chromatography.

### 3.6. Direct Affinity Purification of Chitosanases from Marine Bacteria

In our previous study, sixty-two strains of marine bacteria with chitosan degradation ability were isolated. Among these, twenty-three strains produced chitosanase, nine of which showed high chitosanase activity (>50 U/mL). In this study, the nine bacterial strains with high chitosanase activity were chosen as target strains in affinity purification of chitosanase using the CHDS-based resins. The bacteria were cultured in a medium (containing 30 g/L NaCl, 3 g/L MgSO_4_·7H_2_O, 0.2 g/L CaCl_2_, 0.1 g/L KCl, 0.02 g/L FeSO_4_, 1.5 g/L Na_2_HPO_4_, 1 g/L NaH_2_PO_4_, and 2 g/L chitosan) at 25 °C for 48 h on a rotary shaker (speed, 150 rpm). After that, cultures were centrifuged at 10,000× *g* for 15 min to remove strains. Approximately 500 mL of supernatants was loaded onto a 10 mL pre-equilibrated column. Washing buffers (0.1 M Tris-HCl buffer with 100 mM NaCl, pH 8.0) were used to remove the uncombined proteins. After that, elution buffers (0.1 M acetic acid buffer, pH 5.6, 0.8 M NaCl) were used for eluting the target protein. The flow rate was 5.0 mL/min.

### 3.7. Assay of Enzyme Activity

The 3,5-dinitrosalicylic acid (DNS) method was used for assay of the enzyme activity of chitosanase. Briefly, 100 µL of enzyme was mixed with 900 µL of chitosan substrate (0.3% *w/v*, prepared in 50 mM sodium acetate buffer, pH 6.5). Reaction solution was incubated at 40 °C in a water bath for 10 min. Immediately, 750 µL of DNS solution was added into the reaction solution. After that, the mixtures were heating at 100 °C for 10 min. The reaction mixture was cooled down and then centrifuged at 10,000 × *g* for 2 min to remove the remaining insoluble chitosan. The reducing sugars in the supernatant were analyzed at 520 nm. Each reaction was carried out in triplicate; standard deviation were calculated and used for analysis. D-glucosamine was used as standard. One unit of enzyme activity was defined as the amount of enzyme that releases 1 µmol d-glucosamine-equivalent reducing sugars per minute under the assay conditions.

### 3.8. Analysis of Protein Purity

Protein purity was determined by SDS-PAGE analysis. The purity of the purified chitosanase was estimated based on the intensity of the protein band using Gelpro Analyzer 3.2, a commonly used gel imaging analysis system. HPLC analysis was conducted using Agilent 1260, equipped with a TSK3000SW column (Tosoh Co., Tokyo, Japan), wherein protein was monitored by absorbance at 280 nm [27]. The mobile phase was 0.1 M PBS, pH 6.7, 0.1 M Na_2_SO_4_, 0.05% NaN_3_. The flow rate was 0.6 mL/min.

## 4. Conclusions

In this study, a highly efficient affinity resin designed for chitosanase purification was synthesized and characterized. Among other purification protocols, the synthesized resins using CHDS to couple with Sepharose 6B resin via cyanuric chloride spacer were used in the direct purification of native chitosanase without any tags from bacterial culture. This protocol has several significant advantages, for instance, higher purity, fewer steps, and better activity recovery. Coupled with accessible reagents, efficacy, and time-saving procedures, this efficient affinity purification protocol can be a potentially important tool for screening native chitosanases that possible have unique characteristics.

## Figures and Tables

**Table 1:** Ligand densities, desorption constant (*K*_d_) and theoretical maximum absorption (*Q*_max_) analysis of the affinity media.

Ligands	Spacer Arms	Ligand Density (μmol/mL)	*K*_d_ (μg/mL)	*Q*_max_ (mg/g)
Glucoamine	Cyanuric chloride	20.9	88.5	10.6
CHTS ^a^	Cyanuric chloride	20.9	20.7	24.7
CHDS ^b^	Cyanuric chloride	20.9	16.4	30.9
CHDS	5-atom spacer	41.8	24.2	24.4
CHDS	10-atom spacer	27.8	38.8	20.8

^a^ CHTS represents chitosan trisaccharides; ^b^ CHDS represents chitosan disaccharides.

**Table 2:** Comparison of traditional, CHDS-based, and immobilized metal affinity chromatography (IMAC)affinity purification methods for three different chitosanases.

Enzymes	Purification Method	Activity Recovery (%)	Protein Purity (%)	Specific Activity (U/mg)
CsnOU01 (GH46)	CHDS-based protocol ^a^	64.1	97.8	356.8
	Traditional protocol ^c^	28.2	94.5	358.5
	IMAC protocol ^b^	71.8	95.6	306.7
Csn (GH75)	CHDS-based protocol	45.2	96.3	664.6
	Traditional protocol ^d^	10.5	93.2	682.7
	IMAC protocol	60.7	96.4	592.3
ChoA (GH80)	CHDS-based protocol	40.8	97.1	851.4
	Traditional protocol ^e^	9.2	89.6	847.6
	IMAC protocol	63.4	90.3	727.8

^a^ In the CHDS-based affinity purification protocol, enzymes without 6× his-tag were purified by CHDS-based medium; ^b^ in the Ni-NTA affinity purification protocol, enzymes with 6×his-tag were purified by Ni-NTA medium; ^c^ in the traditional protocol, CsnOU01 without 6×his-tag was purified by five steps, including ultrafiltration, ammonium sulfate precipitation, desalting, anion-exchange, and gel-filtration chromatography; ^d^ the traditional purification protocol of Csn was composed of three steps, including ammonium sulfate precipitation, desalting, and anion-exchange chromatography; ^e^ the traditional purification protocol of ChoA was composed of six steps, including ammonium sulfate precipitation, hydrophobic chromatography, desalting, anion-exchange chromatography, and two steps of gel-filtration chromatography.

**Table 3:** Affinity purification of chitosanases direct from bacterial supernatant.

Number	Bacterium	Activity Recovery (%)	Protein Purity (%)
1	*Bacillus* sp. QD08	49.2	95.4
2	*Bacillus* sp. QD102	40.6	96.2
3	*Bacillus* sp. QD72	41.1	98.1
4	*Paenibacillus* sp. QD03	10.5	92.2
5	*Mitsuaria* sp. QD129	10.7	90.5
6	*Mitsuaria* sp. QD130	39.5	96.7
7	*Renibacterium* sp. QD52	20.3	90.1
8	*Serratia* sp. QD07	12.7	97.8
9	*Flavobacterium* sp. QD28	11.6	91.9

## Supplementary Materials

The following are available online at http://www.mdpi.com/1660-3397/17/1/68/s1, Table S1: Different loading and elution condition on the recovery yield and specific activity of CsnOU01. Table S2: The sum of traditional protocol for three different chitosanase.
