# Supplementary material for: Design and Synthesis of a Chitodisaccharide-Based Affinity Resin for Chitosanases Purification
Source: Mar Drugs. 2019 Jan 21;17(1):68. doi: 10.3390/md17010068 (PMC6356299; doi:10.3390/md17010068)
Supplement: Supplementary file 1 [file marinedrugs-17-00068-s001.pdf]

Supplementary Material

Table S1. Different loading and elution condition on the recovery yield and specific activity of CsnOU01.

Loading condition	Elution condition	Recovery yield (%)	Specific activity (U/mg)
0.1 M Tris-HCl buffer, pH 8.0, 0.1 M NaCl	0.1 M acetic acid, pH 5.4, 0.8 M NaCl	64.1	356.8
0.1 M Tris-HCl buffer, pH 8.0, without NaCl	0.1 M acetic acid, pH 5.4, 0.8 M NaCl	69.8	304.2
0.1 M Tris-HCl buffer, pH 8.0, 0.2 M NaCl	0.1 M acetic acid, pH 5.4, 0.8 M NaCl	48.6	358.2
0.1 M Tris-HCl buffer, pH 8.6, 0.1 M NaCl	0.1 M acetic acid, pH 5.4, 0.8 M NaCl	57.6	349.8
0.1 M phosphate buffer, pH 7.6, 0.1 M NaCl	0.1 M acetic acid, pH 5.4, 0.8 M NaCl	34.7	305.5
0.1 M Tris-HCl buffer, pH 8.0, 0.1 M NaCl	0.1 M acetic acid, pH 5.4, 1 M NaCl	63.7	357.2
0.1 M Tris-HCl buffer, pH 8.0, 0.1 M NaCl	0.1 M acetic acid, pH 5.4, 0.6 M NaCl	60.4	297.1
0.1 M Tris-HCl buffer, pH 8.0, 0.1 M NaCl	0.1 M acetic acid, pH 4.0, 0.8 M NaCl	37.1	357.1
0.1 M Tris-HCl buffer, pH 8.0, 0.1 M NaCl	0.1 M acetic acid, pH 6.0, 0.8 M NaCl	47.5	294.2

Table S2. The sum of traditional protocol for three different chitosanase.

	Steps	Recovery yield (%)	Specific activity (U/mg)
CsnOU01 (5 steps)	0. crude enzyme of CsuOU01	100	81.2
	1. ultrafiltration	94.3	126.7
	2. 60% ammonium sulfate precipitation	86.7	168.7
	3. desalting	84.3	176.7
	4. anion-exchange (DEAE) chromatography	55.4	279.4
	5. gel-filtration (Superdex 75) chromatography	28.2	358.5
Csn (3 steps)	0. crude enzyme of Csn	100	107.6
	1. 60% ammonium sulfate precipitation	75.4	167.1
	2. desalting	67.9	180.4
	3. anion-exchange (DEAE) chromatography	10.5	682.7
ChoA (6 steps)	0. crude enzyme of ChoA	100	36.9
	1. 40% ammonium sulfate precipitation	89.5	72.4
	2. hydrophobic chromatography	57.6	123.5
	3. desalting	56.7	130.5
	4. anion-exchange (DEAE) chromatography	46.9	208.1
	5. gel-filtration (Superdex 75) chromatography	18.6	470.6
	6. gel-filtration (Superdex 200) chromatography	9.2	847.6
